# Characterizing viscoelastic properties of human melanoma tissue using Prony series

**DOI:** 10.3389/fbioe.2023.1162880

**Published:** 2023-04-06

**Authors:** Seungman Park, Anna L. Chien, Isabelle D. Brown, Jingchun Chen

**Affiliations:** ^1^ Department of Mechanical Engineering, University of Nevada, Las Vegas, NV, United States; ^2^ Department of Mechanical Engineering, Johns Hopkins University, Baltimore, MD, United States; ^3^ Institute for NanoBio Technology, Johns Hopkins University, Baltimore, MD, United States; ^4^ Department of Dermatology, Johns Hopkins University School of Medicine, Baltimore, MD, United States; ^5^ Nevada Institute of Personalized Medicine, University of Nevada, Las Vegas, NV, United States

**Keywords:** human melanoma tissue, skin cancer, viscoelastic property, stress relaxation, Prony series, shear modulus, relaxation time, viscosity

## Abstract

Melanoma is the most invasive and deadly skin cancer, which causes most of the deaths from skin cancer. It has been demonstrated that the mechanical properties of tumor tissue are significantly altered. However, data about characterizing the mechanical properties of *in vivo* melanoma tissue are extremely scarce. In addition, the viscoelastic or viscous properties of melanoma tissue are rarely reported. In this study, we measured and quantitated the viscoelastic properties of human melanoma tissues based on the stress relaxation test, using the indentation-based mechanical analyzer that we developed previously. The melanoma tissues from eight patients of different ages (57–95), genders (male and female patients), races (White and Asian), and sites (nose, arm, shoulder, and chest) were excised and tested. The results showed that the elastic property (i.e., shear modulus) of melanoma tissue was elevated compared to normal tissue, while the viscous property (i.e., relaxation time) was reduced. Moreover, the tissue thickness had a significant impact on the viscoelastic properties, probably due to the amount of the adipose layer. Our findings provide new insights into the role of the viscous and elastic properties of melanoma cell mechanics, which may be implicated in the disease state and progression.

## 1 Introduction

Melanoma is one of the deadliest cancer diagnoses because it rapidly metastasizes and spreads to other parts of the body, such as the brain, skin, or lung (Potrony et al., 2015; Kurtansky et al., 2022; Switzer et al., 2022). As such, melanoma is responsible for over 75% of skin cancer deaths, although it accounts for only approximately 1% of skin cancers (Caulfield and Kluger, 2022). The American Cancer Society recently estimated that about 97,610 people are expected to be diagnosed with new melanomas and about 7,990 of those people will die in 2023 (American Cancer Society, 2023). Since the 5-year survival rates for early- and late-stage (i.e., distant metastasis) melanoma are 99% and 32%, respectively, it is extremely crucial to detect melanoma as early as possible.

Currently, standardized methods to diagnose melanoma include physical and imaging examinations by dermatologists and skin biopsy. However, it can be incredibly difficult to distinguish melanoma from common moles or dysplastic nevi, or *vice versa* (Cassileth et al., 1986; Mitsui et al., 2016). Hence, it is essential to also find additional biomarkers and use well-established biomarkers for accurately evaluating melanoma for early detection, thus improving survival rates. Previous studies have shown that the mechanical properties of tumor tissue are significantly altered compared to those of normal tissue (Margueritat et al., 2019; Ishihara and Haga, 2022). More specifically, mechanical properties (e.g., stiffness or Young’s modulus) of many solid tumors, such as mammary (Paszek et al., 2005; Wang and Larin, 2013; Hajjarian et al., 2021), glioblastoma (Miroshnikova et al., 2016; Tao et al., 2021; Bhargav et al., 2022), liver (Mueller, 2010), pancreatic (Shi et al., 2015; Itoh et al., 2016), lung (Miyazawa et al., 2018), ovarian (Mieulet et al., 2021), bladder (Ghasemi et al., 2020), and skin tumors (Troyanova-Wood et al., 2019), were observed to have significantly increased compared to those of normal and healthy tissues.

Despite a myriad of studies to characterize the mechanical properties of different types of tumor tissues, data on the mechanical or viscoelastic properties of human melanoma skin tissues are still scarce. Moreover, there are a very limited number of studies on measuring or comparing the viscous properties, such as relaxation time or viscosity, between normal and melanoma skin tissues, compared to the elastic properties. Quantifying the mechanical and viscoelastic properties of tumor tissues can allow us to understand the underlying mechanisms of cancer development, thereby broadening our knowledge of cancer biology (Madsen and Cox, 2017).

Motivated by this fact, we measured and quantitated the viscoelastic properties of human melanoma tissues based on the stress relaxation test using the indentation-based mechanical analyzer that we developed previously (Park et al., 2019). The melanoma tissues from eight patients of different ages (57–95), genders (male and female patients), races (White and Asian), and sites (nose, arm, shoulder, and chest) at the Johns Hopkins University Hospital were excised and tested. In brief, we applied a constant displacement on the tissue and recorded the resulting force magnitudes. Subsequently, the measured force magnitudes were converted to shear relaxation moduli through theoretical and mathematical models. Lastly, the shear relaxation moduli were the curve fits for a linear viscoelastic model using the Prony series, and the shear modulus, relaxation time, and viscosity were obtained. In addition, to investigate the effect of test environments, such as humidity or moisture, we measured and compared the viscoelastic properties of melanoma tissues submerged in two different fluid conditions, air and liquid. We also examined if the tissue thickness is related to the viscoelastic properties.

## 2 Materials and methods

### 2.1 Human sample preparation and extraction protocol

**FIGURE 1:**
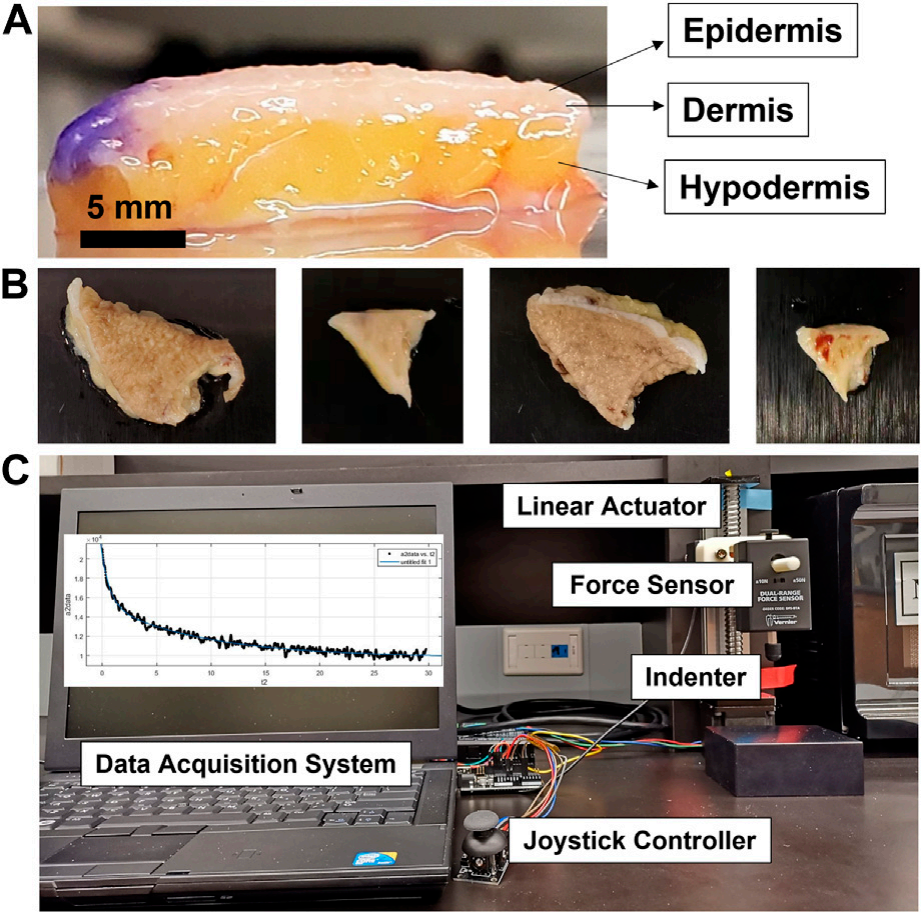
Experimental schematic representation for the characterization of the viscoelastic properties of human skin tissue. **(A)** Melanoma skin tissues displaying epidermis, dermis, and hypodermis layers. **(B)** Different-sized and shaped *ex vivo* melanoma tissues. **(C)** Experimental set-up for the stress-relaxation test using the indentation-based mechanical analyzer composed of the linear actuator, force sensor, indenter, data acquisition system, and joystick controller.

Human melanoma tissues in different sizes and shapes from eight patients were collected during Mohs micrographic surgery at the Johns Hopkins Department of Dermatology (Figures 1A,B). The fresh samples were placed in a saline solution at 4°C before the tests. All the tests were carried out within 48 h after the excision of the tissues. The samples were de-identified, but information was recorded with each sample, including the age, race, sex, and anatomical location.

### 2.2 Measurement of the viscoelastic properties using the indentation-based mechanical analyzer

Viscoelastic properties were measured through a stress relaxation test using the indentation-based mechanical analyzer developed in the laboratory (Figure 1C). The device was calibrated as previously reported (Park et al., 2019). The tissue samples were placed onto the support and the semi-spherical indenter (6.2 mm in radius) combined with a force sensor; it was lightly pressed into the sample by adjusting the joystick displacement controller. After applying constant deformation (0.5–1 mm) to the sample with the indenter, force magnitudes were recorded for 100 s using the force sensor (Dual-Range Force Sensor). The sampling rate of the force data and force resolution were 50 samples/s and 10 mN, respectively. Four of the samples were measured one day, while the other four samples were measured another day. Each tissue sample was tested at three different locations.

### 2.3 Data processing and analysis

There are three steps to derive the viscoelastic properties (i.e., shear modulus, relaxation time, and viscosity). First, the measured force data (*F*) are converted to the shear relaxation modulus (*G*) (Argatov, 2013), given as follows:

$$G=\frac{3F}{16\delta \sqrt{R\delta }},$$

where *G*, *F*, *R*, and *δ* are the shear relaxation modulus, force, radius of the indenter, and indentation depth, respectively. Subsequently, the non-linear curve fit was applied to the data using the Prony series given by the following:

$$G\left(t\right)=G_{\infty }+G_{1}e^{\left(-\frac{t}{\tau_{1}}\right)}+G_{2}e^{\left(-\frac{t}{\tau_{2}}\right)},$$

where $G_{\infty }$ denotes steady-state stiffness, $G_{1}$ and $G_{2}$ denote the shear modulus of the first and second terms, $\tau_{1}$ and $\tau_{2}$ represent the relaxation time of the first and second terms, and t is the time. The shear modulus was directly fitted to the Prony series.

The instantaneous shear modulus (*G*_Ins_) and equivalent viscosity (*μ*_Eq_) can be calculated using the following relations (Tupin et al., 2016):

$$G_{Ins}=G_{\infty }+G_{1}+G_{2},$$

$$\mu_{Eq}=2\left(1+\nu \right)\frac{{\left(G_{1}+G_{2}\right)}^{2}}{\left[G_{1}/\tau_{1}+G_{2}/\tau_{2}\right]}.$$

### 2.4 Statistical analysis

Each test group was repeated at least three times (n ≥ 3), and the results were presented as mean ± standard error. One-way ANOVA was carried out for the statistical analysis, and the differences were considered statistically significant when the *p*-value was less than 0.05.

## 3. Results

### 3.1 Comparison of viscoelastic properties of air- and liquid-submerged tissues

**FIGURE 2:**
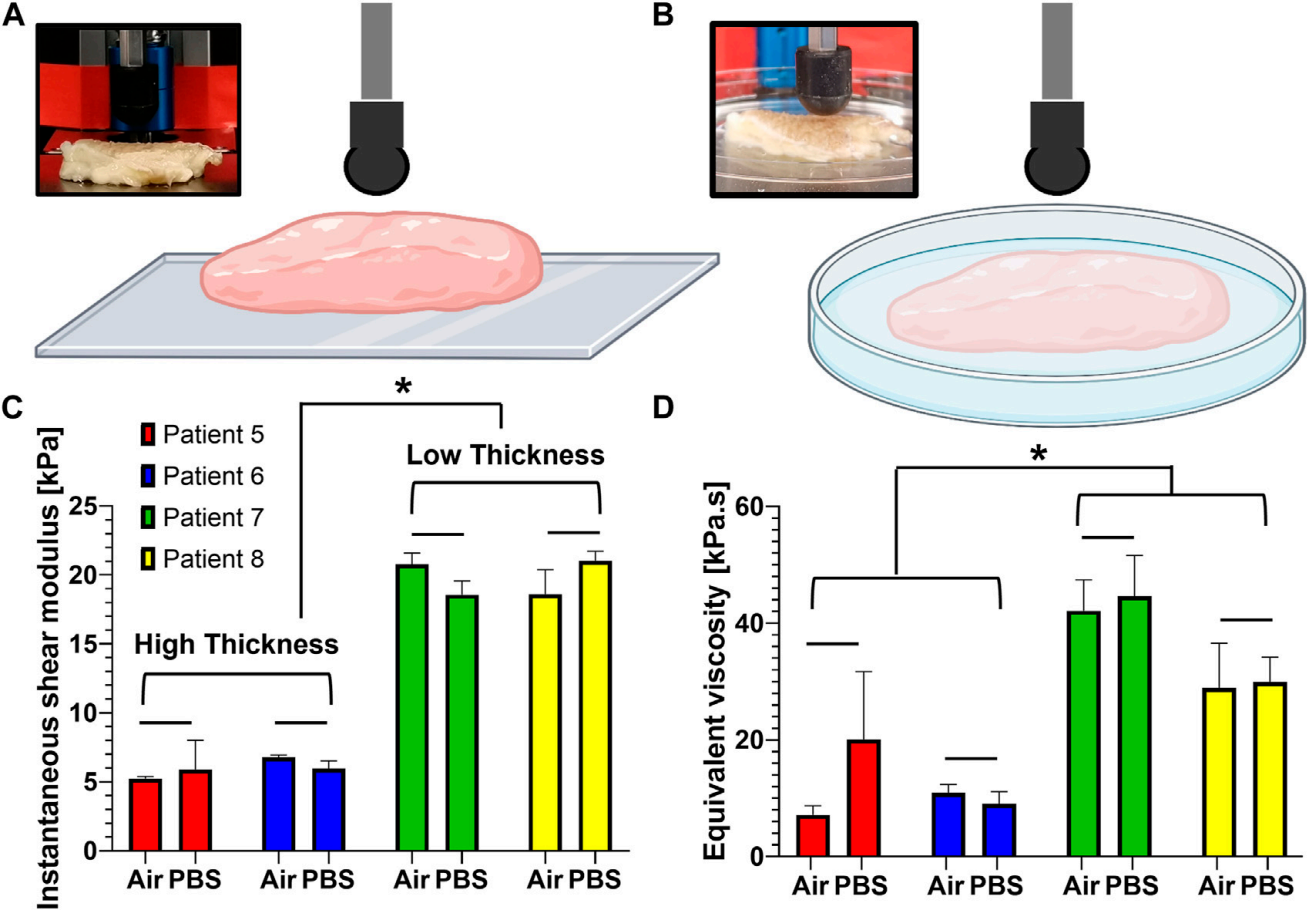
Comparison of the viscoelastic properties obtained from human melanoma tissues in air and PBS. **(A, B)** Schematic representation for the measurement of the viscoelastic properties between two submersion modes: air **(A)** and PBS **(B)**. **(C)** Instantaneous shear moduli obtained from the samples when submerged in air and PBS. **(D)** Equivalent viscosities obtained from the samples when submerged in air and PBS. Data are shown as mean ± standard error, and each test group was repeated at least three times. *p < 0.05; bar (−): not significant.

We first checked if there is any difference in the viscoelastic properties between air- and liquid-submerged melanoma skin tissues. Four patient samples were randomly selected and evaluated using two test conditions, air *vs.* phosphate-buffered saline (PBS) (Figures 2A,B), and their results were compared. The results revealed no statistically significant difference in both the instantaneous shear modulus (air and PBS: 5.23 ± 0.12 kPa and 5.89 ± 1.73 kPa for patient 5; 6.79 ± 0.12 kPa and 5.98 ± 0.44 kPa for patient 6; 20.77 ± 0.67 kPa and 18.57 ± 0.81 kPa for patient 7; and 18.60 ± 1.45 kPa and 21.03 ± 0.56 kPa for patient 8) (Figure 2C) and equivalent viscosity (air and liquid: 7.16 ± 1.28 kPa·s and 20.09 ± 9.49 kPa·s for patient 5; 10.94 ± 1.17 kPa·s and 9.06 ± 1.68 kPa·s for patient 6; 42.14 ± 4.31 kPa·s and 44.61 ± 5.71 kPa·s for patient 7; and 28.97 ± 6.20 kPa·s and 29.9 ± 3.51 kPa·s for patient 8) (Figure 2D) between the two measurement conditions.

### 3.2 Effects of tissue thickness on the viscoelastic properties

We also examined if tissue thickness impacts the viscoelastic properties (Figures 2C,D). The thickness of the tissues shows 10, 14, 5, and 6 mm for patients 5, 6, 7, and 8, respectively. The viscoelastic properties were found to be highly dependent on the thickness. The tissues with a lesser thickness tend to have a higher instantaneous shear modulus than those of low thickness (Figure 2C). The same is true for the equivalent viscosity (Figure 2D). More specifically, the low-thickness group (low thickness) yielded an instantaneous shear modulus (19.74 ± 0.17 kPa), approximately 3.3 times higher than that (5.97 ± 0.28 kPa) of the high-thickness group (high thickness). When it comes to the viscous property, the equivalent viscosity (36.41 ± 0.54 kPa·s) of the low-thickness group was approximately 3.1 times higher than that of the high-thickness group (11.81 ± 2.48 kPa·s).

### 3.3 Comparison of the viscoelastic properties of human normal and melanoma skin tissues

The viscoelastic properties of melanoma tissues from eight patients are summarized in Table 1. The values for *G*_*∞*_, *G*_*1*_, *G*_*2*_, *τ*_*1*_, and *τ*_*2*_ are the mean values of the data measured at least three times, and *G*_*ins*_ and *μ*_*eq*_ are calculated based on the mean values. Results showed that the maximum and minimum instantaneous shear modulus (*G*_*ins*_) are 58.27 kPa for patient 2 and 5.23 kPa for patient 5, respectively. The maximum and minimum equivalent viscosity (*μ*_*eq*_) are 141.472 kPa·s for patient 2 and 7.16 kPa·s for patient 5.

**TABLE 1:** Viscoelastic properties of human melanoma tissues from eight patients.

Patient	*Dimension [mm^3^]	*G*_*∞*_ [kPa]	*G*_*1*_ [kPa]	*G*_*2*_ [kPa]	*τ*_*1*_ [s]	*τ*_*2*_ [s]	*G*_*ins*_ [kPa]	*μ*_*eq*_ [kPa·s]
1	30 × 40 × 4	8.06	17.20	11.70	0.55	7.39	36.98	76.02
2	20 × 20 × 3	4.05	34.0	20.20	0.580	5.47	58.27	141.47
3	40 × 30 × 6	3.58	8.72	6.26	0.56	2.00	18.56	11.01
4	20 × 20 × 5	14.79	10.92	13.65	1.29	2.64	39.37	40.61
5	40 × 40 × 10	3.36	0.90	0.96	0.67	12.98	5.22	7.15
6	50 × 40 × 14	4.51	1.03	1.24	0.77	13.77	6.79	10.93
7	20 × 20 × 5	9.88	5.34	5.53	0.68	8.62	20.76	42.13
8	20 × 20 × 6	10.70	4.11	3.77	0.69	9.13	18.59	28.96

*Dimension denotes width × height × thickness.

**FIGURE 3:**
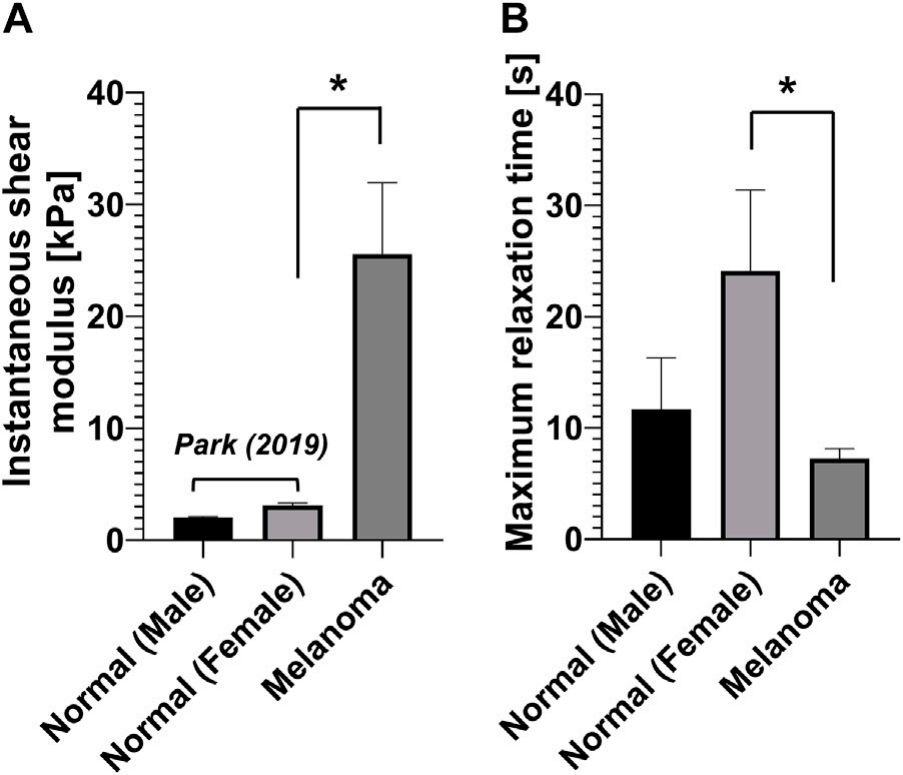
Viscoelastic properties of human normal (Park et al., 2019) and melanoma skin tissues. **(A)** Instantaneous shear modulus. **(B)** Maximum relaxation time.

We compared the measured instantaneous shear modulus, relaxation time, and equivalent viscosity of human melanoma tissues with those of normal tissues measured using the same device (i.e., indentation-based mechanical analyzer) previously reported (Park et al., 2019) (Figure 3). The results exhibited that the viscoelastic properties are markedly different in the melanoma tissues from normal ones. For example, the instantaneous shear modulus of the melanoma tissues (25.58 ± 5.96 kPa) was approximately 12.6 times and 8.2 times greater than those of the normal tissues for male (2.03 ± 0.07 kPa) and female patients (3.12 ± 0.21 kPa), respectively (Figure 3A). In contrast, the maximum relaxation time of the melanoma tissues (7.24 ± 0.88 s) was significantly lower than that of the normal tissues of male (11.69 ± 4.45 s) and female patients (24.12 ± 7.01 s) (Figure 3B). Intriguingly, the equivalent viscosity of the melanoma tissues (22.3 ± 4.51 kPa·s) was not different from that of the normal tissues of female patients (21.2 ± 9.09 kPa·s).

## 4 Discussion

In the current study, we characterized both the elastic and viscous properties of human melanoma skin tissues from eight patients at the Johns Hopkins University Hospital and compared them with those of normal skin tissues measured previously (Park et al., 2019). The results showed that there is an elevated elastic property (i.e., shear modulus) of melanoma tissue than that of normal tissue but a reduced viscous property (i.e., relaxation time). This result aligns with the previous findings that tumor tissues exhibit a greater stiffness compared to normal tissues. These tumors include colon (Bauer et al., 2020), breast (Watson et al., 2021), liver (Masuzaki et al., 2007), and pancreatic tumors (Ishihara and Haga, 2022). Additionally, the study found that tissue thickness was negatively correlated with both the elastic and viscous properties. In contrast, no difference was found in the viscoelastic properties between air- and PBS-immersed tissues.

The mechanical property of cells and tissues can be used as a biosensor to represent a state of disease or aging. To characterize the mechanical properties of these biological materials, diverse techniques have been utilized, such as AFM (Cross et al., 2007; Garcia et al., 2020), stretcher (Trepat et al., 2007), micropipette aspiration (Discher et al., 1998), or optical tweezers (Guck et al., 2001). Studies have demonstrated that cancer cells show a lower elastic modulus or more stiffness than the normal cells (Cross et al., 2007; Lekka et al., 2012). In addition, metastatic cancer cells are less stiff and more deformable than non-metastatic cancer cells (Liu et al., 2020). Another study suggested that plasticity, the ability of materials to adjust their mechanical properties to external conditions, is a more significant marker for tumor malignancy, compared to stiffness or other mechanical properties (Weder et al., 2014). At the tissue level, it has also been shown that melanoma tissues are stiffer than normal tissues, which is associated with promoting the proliferation and invasiveness of melanoma cells (Staunton et al., 2016; Andrlová et al., 2017; Li et al., 2019). The experimental investigation by Troyanova-Wood et al. exhibited that melanoma tissues have a greater stiffness than the normal surrounding tissues through elasticity-specific Brillouin spectroscopy (Troyanova-Wood et al., 2019). In that study, they observed that the average Brillouin shifts are 8.55 GHz for melanoma tissues and 7.97 GHz for healthy tissues. However, a contradictory result also exists. Park et al. characterized the mechanical properties of formalin-fixed paraffin-embedded (FFPE) human skin tissues and discovered that the Young’s modulus of normal skin (6–8 MPa) is higher than that of melanoma tissue (4–6 MPa) by 17.5% on average (Park et al., 2020). Moreover, both storage and loss modulus for normal skin tissues tended to be higher than melanoma skin tissues over most of the loading frequency ranges.

The increase in the mechanical properties of melanoma tissue could be mainly due to the accumulation of collagen or other molecules, elevated contraction, and cross-linking. On the other hand, the viscous behavior might be diminished due to the increased portions of solid components and molecules, such as collagen, the resulting lower amount or portion of interstitial fluid. However, how viscous properties change in the tumor tissue remains unclear. Hence, more studies are required to elucidate the relationship between tumor and viscous properties.

It has been suggested that tissue thickness can be one of the significant factors impacting magnitudes of the mechanical or viscoelastic properties of tissue. Griffin et al. excised human skin tissues of five different skin sites: forehead, submandibular neck, temporoparietal neck, postauricular mastoid, and forearm (Griffin et al., 2017). The tissue thickness was measured using electronic calipers and was shown to be 1.4 mm for the forehead, 1.39 mm for the temporoparietal region, 1.23 mm for the postauricular mastoid, 1.19 mm for the forearm, and 0.87 mm for submandibular neck. However, the measured elastic modulus was negatively correlated with the thickness, revealing 1.28 MPa for the submandibular skin, 1.03 MPa for the forearm, 0.86 MPa for the postauricular mastoid, 0.65 MPa for the temporoparietal skin, and 0.33 MPa for the forehead. The different thicknesses of excised melanoma tissues may be primarily due to the different amounts of adipose tissue, based on the observation that melanoma tissues of higher thicknesses tend to have a greater amount of the adipose layer (Figure 1), where the adipose layer is mainly dominant and plays a key role in determining mechanical or viscoelastic properties of the whole tissue, compared to dermis and epidermis layers (Groves et al., 2012; Wei et al., 2022).

The thickness effect strongly depends on the ratio of the contact radius to the layer thickness. Previous studies have shown that the effect of tissue thickness on the time-dependent mechanical response to indentation by the spherical indenter is crucial, whereas little effect in the flat-ended indentation test is observed (Argatov et al., 2013). The thickness effect on the instantaneous response can be estimated based on the solution of the spherical indentation of an elastic layer (Hayes et al., 1972).

In this study, we focused on testing the area within 10 mm of tumor tissue to make sure that the viscoelastic properties of the tumor tissue and not the healthy tissue were measured. The tumor itself, tumor microenvironment, and the other surrounding area are all affected by proinflammatory factors and molecules released from tumor cells (Zhang et al., 2021). For example, tumor cells secrete diverse growth factors, proinflammatory cytokines, glycoproteins, enzymes, and exosomes. These molecules significantly change biochemical or physical properties of tumors and promote the progression and metastasis of cancer (Anderson and Simon, 2020).

It is found that different methods or techniques result in different magnitudes of mechanical properties. Researchers have measured the stiffness values of separate layers, such as the stratum corneum, epidermis, dermis, and hypodermis. The reported mechanical properties are significantly different, up to several orders of magnitude. The difference may come from the different techniques, theoretical models and experimental conditions (Liang and Boppart, 2010; Van Kuilenburg et al., 2013; Chen et al., 2014; Panchal et al., 2019).

We conducted a non-harmonic experiment using the time-dependent viscoelastic model. However, the viscoelastic properties of viscoelastic materials are known to be highly dependent on frequency. For instance, viscoelastic materials tend to have higher stiffness at high frequencies than low frequencies (McCraw et al., 2021). These harmonic quantities include the storage modulus, representing the elastic portion and loss modulus representing the viscous portion. Thus, further harmonic (i.e., frequency-dependent) studies are warranted to better understand the mechanical or viscoelastic behavior of human melanoma skin tissues (Parvini et al., 2022).

There are some key limitations to be addressed in future studies. First, we did not take into account effects of several factors, such as the gender, age, or body site of melanoma, which may influence the viscoelastic properties. In this study, the viscoelastic properties of human melanoma skin tissues were averaged, irrespective of the measurement sites, age or sex. The current study is based on the rationale that the mechanical properties only vary based on the presence of cancer but not in the presence of other factors. For example, the experimental results by Jeon et al. showed that the elastic modulus highly varies depending on the normal, benign nevus, or melanoma tissues but not on the measurement sites nor age and sex (Jeon et al., 2022). On the other hand, many studies have reported that the mechanical properties are also highly dependent on the these factors addressed previously. For instance, Park et al. observed that the Young’s modulus of the anterior and posterior skin tissue is significantly higher than that of forearm tissue through indentation (Park et al., 2019). An age-related decrease, by 40% from the age of 18–40 to 60–80, in stiffness, was also observed in the papillary and reticular dermis (Lynch et al., 2022). Second, in the current study, we measured the viscoelastic properties of *ex vivo* human melanoma skin tissues, which is different from the previously measured condition of *in vivo* normal skin tissues, neglecting the possibility that the different states (i.e., *in vivo*, *in situ*, *ex vivo*, and *in vitro*) of the samples may affect the results of the viscoelastic properties. For example, previous research revealed that skin elasticity tends to be higher *in vivo* than that in *in situ* conditions (Wei et al., 2022). Another study investigated by Groves et al. identified significant differences in the mechanical properties between *in vivo* and *ex vivo* tissues of human and mouse skin (Groves et al., 2012). Taken together, more systematic research is needed to understand the combined and profound effects of diverse factors, including the presence of cancer, sample state, gender, age, and body site of melanoma, on the viscoelastic properties.

## Data Availability

The original contributions presented in the study are included in the article/Supplementary Material; further inquiries can be directed to the corresponding author.
